# Hematopoietic stem cell transplantation following invasive mold infection in chronic granulomatous disease: Insights from a case series and literature review

**DOI:** 10.70962/jhi.20250164

**Published:** 2026-03-05

**Authors:** Hugo Bes-Berlandier, Olivier Paccoud, Benedicte Neven, Marie Elisabeth Bougnoux, Emilie Sitterlé, Ambroise Marcais, Morgane Cheminant, Despina Moshous, Rinzine Sammut, Pierre Simon Rohrlich, Nizar Mahlaoui, Olivier Lortholary, Felipe Suarez, Fanny Lanternier

**Affiliations:** 1 Université Paris Cité, Service de Maladies Infectieuses et Tropicales, Hôpital Necker–Enfants Malades, Paris, France; 2 Pediatric Immuno-Hematology and Rheumatology Unit, Necker-Enfants University Hospital, Assistance Publique-Hôpitaux de Paris, Paris, France; 3 Imagine, U1168, Immunogenetics of Autoimmune Diseases, Paris, France; 4 Université Paris Cité, Service de Mycologie-Parasitologie, Hôpital Necker–Enfants Malades, Paris, France; 5 Université Paris Cité, Service D’hématologie Adultes, Hôpital Necker–Enfants Malades, Assistance Publique-Hôpitaux de Paris, Paris, France; 6 Service D’hématologie Clinique, Centre Hospitalier Universitaire de Nice, Nice, France; 7 French National Reference Center for Primary Immunodeficiencies, Necker-Enfants University Hospital, Assistance Publique-Hôpitaux de Paris, Paris, France; 8 Centre National de Référence des Mycoses Invasives et Antifongiques, Institut Pasteur, Paris, France

## Abstract

Chronic granulomatous disease (CGD) is an inborn error of immunity leading to severe invasive mold infections (IMIs). Questions remain regarding the safety of hematopoietic stem cell transplantation (HSCT) in patients following IMI. We retrospectively collected data from CGD patients undergoing HSCT within 2 years after a diagnosis of probable or proven IMI in France. The primary endpoint was to describe 1-year overall survival (OS). 14 patients were included, with aspergillosis (*n* = 10), mucormycosis (*N* = 2), scedosporiosis, or lomentosporiosis (*n* = 1 each). IMI status at time of HCST included four in complete response, four in partial response (PR), one in stable response (SR), and five with disease progression (DP). 1-year OS was 86 % (12/14). Two patients with scedosporiosis and lomentosporiosis died before engraftment. The remaining patients all achieved engraftment followed by prolonged clinical response of IMI. HSCT appears safe in patients with complete response or PR of IMI and could be a promising curative option for progressive IMI, specifically in those with invasive aspergillosis.

## Teaser

Bes-Berlandier et al. describe the characteristics and clinical outcomes of chronic granulomatosis disease patients undergoing hematopoietic stem cell transplantation (HSCT) within 2 years after an invasive mold infection (IMI) diagnosis. HSCT could be a promising curative option for progressive IMI, specifically in those with invasive aspergillosis.

## Graphical Abstract

**Figure:**
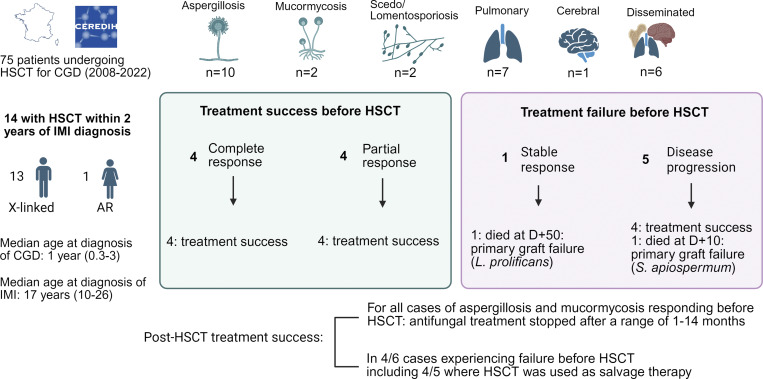

## Introduction

Chronic granulomatosis disease (CGD) is an inborn error of immunity caused by mutations in the genes encoding subunits of the nicotinamide adenine dinucleotide phosphate oxidase complex, leading to impaired neutrophil phagocytic function. Bacterial and fungal infections are the main manifestations of CGD, as well as dysregulated inflammatory responses and granuloma formation affecting various organs (1). Although the prognosis of CGD patients has improved with routine antifungal prophylaxis, invasive mold infections (IMIs) remain the leading cause of infection-related mortality in CGD patients (2). Indeed, the reported lifetime incidence of IMI before the introduction of antifungal prophylaxis ranges from 20% to 46%, with respectively *Aspergillus fumigatus* and *Aspergillus nidulans* as the main organisms isolated (3, 4, 5, 6, 7). In addition, there are increasing reports of azole-resistant aspergillosis and IMI caused by non-*Aspergillus* molds. These often occur as breakthrough infections and/or in patients receiving additional immunosuppressive treatments (8), which requires the development of new therapeutic approaches (4, 9). Hematopoietic stem cell transplantation (HCST) is the only effective long-term treatment for curing immune deficiency, reversing inflammatory lesions, and improving patient survival in CGD patients (10, 11). While recent publications agree on the benefit of offering allogeneic transplantation from childhood onward, in view of its good long-term tolerance, questions remain with regards to the optimal timing of HSCT in patients with current IMI (12, 13). Indeed, while restored phagocytic function after HSCT can contribute to immunological control of aspergillosis, the aplasia induced by this procedure raises questions about its safety in patients with an active or refractory fungal infection.

We therefore sought to describe the characteristics and clinical outcomes of CGD patients undergoing HSCT within 2 years after an IMI diagnosis.

## Results

75 patients with CGD from the French National Reference Center for Primary Immunodeficiencies (CEREDIH) registry underwent HCST between 2008 and 2022. 14 patients met the criteria and were included in the study. Main HSCT characteristics of patients are summarized in Table 1. These 14 patients were older than patients with CGD who received HCST without any documented evidence of IMI (15 years [10, 11, 12, 13, 14, 15, 16, 17, 18, 19, 20, 21, 22, 23, 24, 25, 26, 27, 28, 29, 30] vs. 9 years [4, 5, 6, 7, 8, 9, 10, 11, 12, 13, 14, 15]) (Table S1). Moreover, patients treated with HCST in the setting of IMI more frequently had matched sibling donors (MSDs) and matched unrelated donors (MUDs), while the other groups were more often transplanted with mismatched related haploidentical donors (MMRDs). Interestingly, we found similar 1-year mortality rates for each group (of, respectively, 13% and 14%).

**Table 1.** Characteristics of 14 CGD patients with IMI within 2 years prior to HSCT

​	*N* = 14 (%)
**Sex**	
Male	13 (93)
**CGD characteristics**	​
Median age at diagnosis of CGD (years) [IQR]	1 [0.3–3]
**Genetic pattern**	
X-linked CGD	13 (*93*)
AR	1 (7)
History of inflammatory diseases	5 (36)
Immunosuppressive treatment^a^	6 (43)
**IMI characteristics**	
Median age at diagnosis of IMI (years) [IQR]	15 [10–27]
Infection status at the time of HSCT	​
CR	4 (28.5)
PR	4 (28.5)
SR	1 (7)
DP	5 (36)
Median time between IMI and HSCT (months) [IQR]	10 [6–21]
Median time between IMI and HSCT among patients with partial or stable responses and disease progression (months) [IQR]	7 [4–17]
**Fungal species**	
*Aspergillus* spp	10 (71)
*Mucorales*	2 (14)
*L. prolificans*	1 (7.5)
*S. apiospermum*	1 (7.5)
**Site of infection**	
Lung only	7 (50)
Brain only	1 (7)
Disseminated infection	6 (43)
**HSCT**	
Age at HCST (years)	15 [10–30]
Median year of HSCT	2018 [2015–2019]
**Donor type**	
MSD	6
MUD	3
MMUD	4
MMRD	1
**Stem cell source**	
BM	10 (71)
PBSC	4 (29)
CD34 cells (cells per kg bodyweight) [IQR]	5.9 × 10^6^ [4–9.2]
**Conditioning intensity**	
RTC	1 (7)
RIC	13 (93)
Engraftment	12 (86)
Median time at neutrophil engraftment (days) [IQR]	17 [15–22]
Median time of platelet recovery (days) [IQR]	18 [13–21]
Medial duration of post-HSCT antifungal treatment (months)	4 [3–11]
**Outcome**	
1-year OS	12 (86)
Median DHR at 1 mo after HSCT (%)	89 [74–95]
3-mo median chimerism status (% donor)	98 [94–99]
3-mo clinical and radiological response ^b^	8 (80)
1-year persistent clinical and radiological cure	12 (86)

IQR, interquartile range; BM, bone marrow; RTC, myeloablative reduced toxicity conditioning.

a In the year before the diagnosis of IMI.

b Clinical and radiological evaluation were carried out only in IMI with PR, SR, and DP.

### Characteristics of the 14 patients undergoing HCST after an IMI diagnosis (Tables 1 and 2)

**Table 2.** Individual characteristics of patients undergoing HSCT

Patients	P1	P2	P3	P4	P5	P6	P7	P8	P9	P10	P11	P12	P13	P14
Gender	Male	Male	Male	Male	Male	Male	Male	Male	Female	Male	Male	Male	Male	Male
**CGD**														
Age at diagnosis (years)	1	3	3	1	3	1	1	8	0.1	3	0.1	0.1	0.1	27
Genetic pattern	X-linked CGD	X-linked CGD	X-linked CGD	X-linked CGD	X-linked CGD	X-linked CGD	X-linked CGD	X-linked CGD	AR	X-linked CGD	X-linked CGD	X-linked CGD	X-linked CGD	X-linked CGD
Comorbidity	None	Alloimmunization	Alloimmunization	Alloimmunization	Alloimmunization	No	No	CKD	No	No	No	No	CKD	No
History of inflammatory diseases	None	Liver	CNS, colitis	No	No	No data	No	No: Lung	Yes: Colitis	Yes: Colitis	No	No	No	No
History of previous infections	*Aspergillus* spp	*Staphylococcus * *aureus*	*S. aureus*, *A. fumigatus*	*S. aureus*	*A. fumigatus*	*A. fumigatus*	*A. fumigatus, Serratia marcescens*	*S. aureus*	*S. aureus*	*Aspergillus sydowii*	S. *aureus*	No	*Burkholderia * *multivorans*	S. *aureus*, *A. fumigatus*
Immunosuppressor treatment ^a^	No	Rituximab	Jak inhibitors	Rituximab	Rituximab	CTC	No	No	No data	Adalimumab, AZA, CTC	No	No	No	No
**IMI**														
Age at diagnosis (years)	14	19	38	13	14	14	29	23	0.1	9	0.2	0.1	38	28
Antifungal primary prophylaxis	Itraconazole	Posaconazole	Itraconazole	No data	Itraconazole	Itraconazole	No itraconazole	Posaconazole	No	Posaconazole	No	No	Posaconazole	Posaconazole
Delay between IMI and HSCT (months)^b^	28	12	3	23	4	3	7	19	5	7	25	7	14	22
Fungal species ^c^	*A. fumigatus*	*A. nidulans*	Azole-R *A. fumigatus*	*S. apiospermum*	*A. nidulans*	*L. prolificans*	*A. fumigatus*	Azole-R *A. fumigatus*	*Lichtheimia* spp	*Mucorales* spp	*A. fumigatus*	*A. fumigatus*	*Aspergillus* spp	*A. fumigatus*
Site of infection	Brain^d^	Disseminated: Lung, cardiac, and mediastinal	Lung	Disseminated: Brain^d^ and bones	Disseminated: Lung and bones	Disseminated: Lung, fungemia, and skin	Lung	Disseminated: Lung and thyroid	Lung	Lung	Lung	Lung	Lung	Disseminated: Brain^d^ and lung
Antifungal treatment	Voriconazole and caspofungin	Isavuconazole and caspofungin	LAmb and caspofungin	Voriconazole and caspofungin	Isavuconazole and caspofungin	Voriconazole, LAmB, and terbinafine	Posaconazole	LAmB and caspofungin	LAmB	LAmB	LAmB	LAmB and caspofungin	Isavuconazole	Voriconazole
Additional treatment	Interferon	Granulocyte transfusion^e^	Arterial embolization	Granulocyte transfusion^e^	Granulocyte transfusion^e^	Granulocyte transfusion^e^	No	No	No	No	No	No	No	No
**HSCT**														
Age at HCST	18	20	38	15	14	14	30	25	0.6	10	2	0.8	39	30
HCT-CI score^f^	1	3	1	2	1	1	3	1	2	1	1	1	1	3
Donor type	MUD	MUD	MMUD	MSD	MSD	MMUD	MSD	MMUD	MSD	MMRD	MSD	MUD	MUD	MSD
Stem cell source	BM	PBSC	PBSC	BM	BM	BM	BM	PBSC	BM	BM	BM	BM	PBSC	BM
HLA matching	10/10	10/10	9/10	10/10	10/10	9/10	10/10	No data	10/10	No data	10/10	12/12	10/10	10/10
CD34 cells (cells per kg bodyweight)	3.5 × 10^6^	5.7 × 10^6^	4 × 10^6^	7.2 × 10^6^	9.2 × 10^6^	6.1 × 10^6^	1.7 × 10^6^	5.6 × 10^6^	12 × 10^6^	9.06 × 10^6^	17 × 10^6^	10.9 × 10^6^	12 × 10^6^	4.1 × 10^6^
Conditioning intensity^g^	RIC	*RIC*	RIC	RIC	RIC	RIC	RIC	RIC	RIC	RIC	RIC	RIC	RIC	RTC
Serotherapy	Alemtuzumab	Alemtuzumab	Alemtuzumab	Alemtuzumab	ATG	Alemtuzumab	ATG	Alemtuzumab	ATG	Alemtuzumab	ATG	Alemtuzumab	Alemtuzumab	ATG
GVHD prophylaxis^h^	Ciclosporin + MMF	Ciclosporin + MMF	Ciclosporin + MMF	Ciclosporin + MMF	Ciclosporin + MMF	Ciclosporin + MMF	Ciclosporin + MMF	Ciclosporin + MMF	Ciclosporin + MMF	Ciclosporin + MMF	Ciclosporin + MMF	Ciclosporin + MMF	Ciclosporin + MMF	Ciclosporin
Engraftment^i^	Yes	Yes	Yes	No	Yes	No	Yes	Yes	Yes	Yes	Yes	Yes	Yes	Yes
Time of resolution of aplasia (days post-HSC infusion)	27	14	24	–	13	–	23	15	17	21	16	15	20	15
Time of platelet recovery (days post-HSC infusion)	No data	44	10	–	18	–	17	14	11	22	19	39	19	12
Post-HSCT antifungal treatment time (mo) (% donor)	51	14	4	​	90	–	12	2	1	3	5	2	3	No data
Post-HSCT infection	*Klebsiella* bacteremia	CMV reactivation	BK virus	HSV reactivation	No	Fungemia	No	No	*Klebsiella* bacteremia	No	No	No	*Rhodotorula*	*Clostridium difficile*
**Outcome**														
1-year OS	Alive	Alive	Alive	Dead (day +10 after HSCT)	Alive	Dead (day +50 after HSCT)	Alive	Alive	Alive	Alive	Alive	Alive	Alive	Alive
DHR at 1 mo after HSCT (%)	92	78	96	–	40	–	99	89	94	81	87	95	67	No data
3-mo chimerism status (% donor)	94	99	99	–	94	–	99	98	98	91	96	97	99	99
3-mo clinical and radiological response	Yes	Yes	Yes	No	Yes	No	Yes	Yes	Yes	Yes	–	–	–	–
1-year persistent clinical and radiological cure	Yes	Yes	Yes	No	Yes	No	Yes	Yes	Yes	Yes	Yes	Yes	Yes	Yes

This table includes individual characteristics of patients undergoing HSCT with DP of IMI (patients 1–5), patients undergoing HSCT with SR (patient 6) and PR (patients 7–10) of IMI, and patients undergoing HSCT with CR of IMIs (patients 11–14). CKD, chronic kidney disease; CNS, central nervous system; Azole-R, azole resistant; BM, bone marrow; MMRD, HLA-mismatched related donor; ATG, antithymocyte globulin (thymoglobuline); LAmB, liposomal amphotericin B; MMF, mycophenolate mofetil; GT, granulocyte transfusion; AZA, azathioprine; CTC, corticosteroids; HCT-CI, hematopoietic cell transplantation–specific comorbidity index; RTC, myeloablative reduced toxicity conditioning, BK: Human Polyomavirus 1, ARDS: acute respiratory distress syndrome.

a In the year before the diagnosis of invasive fungal infection.

b Delay between the first microbiological evidence of IMI and the last HSCT.

c Azole-R *A.fumigatus* was defined by a documented resistance to all the triazole.

d Patient 1 underwent a brain biopsy at the onset of the infection to identify the fungal species. After discussion with the neurosurgeons, the brain lesions of patient 4 and patient 14 were deemed inaccessible for biopsy.

e Patient 2 received a granulocytes transfusion three times a wk from month three before HSCT until 3 wk after HCST. Patient 4 received a granulocytes transfusion at the time of IFI diagnosis, but we did not find any information about either frequency of administration or the duration of treatment. Patient 5 received a granulocytes transfusion from 2 mo before HCST until 2 wk before HCST. Patient 6 received a granulocytes transfusion from 2 wk after HCST until death.

f HCT-CI is a validated scoring system that assesses the risks of patients undergoing HSCT.

g Patients received a fludarabine-busulfan based platform including RIC dose (busulfan = 9.6 mg/kg i.v., *n* = 12) and RTC dose (busulfan = 12.8 mg/kg i.v.).

h Ciclosporin was initiated from day 1 before reinfusion and stopped on day 180 after reinfusion. MMF was initiated from the day of reinfusion and stopped on day 100 after reinfusion.

i Graft enhancement was defined by a resolution of aplasia and a bone marrow composition including >50% of cell donor in the chimerism analysis.

13 patients were male with X-linked CGD, and one was a female with autosomal recessive (AR) CGD. The age at first symptom of CGD ranged from 0.3 to 3 years, and the median age at diagnosis of CGD was 1 year. Five patients had a history of inflammatory manifestations associated with CGD (inflammatory bowel disease, *n* = 3, liver, *n* = 1, lung, *n* = 1), and were treated with an immunosuppressive treatment (rituximab *n* = 1; Jak inhibitors, *n* = 1; TNF α inhibitors and corticosteroids, *n* = 1) within the year before HCST. Three patients received rituximab in the context of HLA alloimmunization. Among those with available data, 10 patients were receiving primary antifungal prophylaxis prior to the IMI, specifically with itraconazole (*n* = 5) or posaconazole (*n* = 5). The median age at diagnosis of IMI was 15 years (10, 11, 12, 13, 14, 15, 16, 17, 18, 19, 20, 21, 22, 23, 24, 25, 26, 27) and the median time between IMI and HCST was 10 mo (6, 7, 8, 9, 10, 11, 12, 13, 14, 15, 16, 17, 18, 19, 20, 21). This tended to shorten over the study period and was 22 mo in patients transplanted before 2018 (the median year of HSCT in this cohort) and 7 mo in those transplanted since 2018.

With regards to infection status at the time of HCST, five infections were classified as being in disease progression (DP), one was in stable response (SR), four were in partial response (PR), and four were in complete response (CR).

12 IMI were proven and two were probable. The most frequent mold species was *Aspergillus* spp (*A. fumigatus*, *n* = 7/14, *A. nidulans*, *n* = 2/14, and *Aspergillus* spp, *n* = 1/14), and the remaining were *Lichtheimia* spp (*n* = 1/14), *Mucorales* spp (1/14), *Scedosporium apiospermum* (*n* = 1/14), and *Lomentospora prolificans* (*n* = 1/14). Infection was disseminated in six cases and localized in eight involving the lung (*n* = 7) or brain (*n* = 1). Localized aspergillosis presented as pneumonia in six patients and brain abscesses in one. Of the four patients with disseminated aspergillosis, one experienced a cardiac, mediastinal, and lung involvement; the second a pulmonary and thyroid involvement, while the third and fourth had a pulmonary aspergillosis with two brain microabscesses and a lung and costal involvement, respectively. The two cases of mucormycosis presented as localized pneumonia. The *S. apiospermum* infection was disseminated with a brain abscess and costal osteitis. Finally, the *L. prolificans* infection was also disseminated with pneumonia, fungemia, and skin involvement. Adjunctive therapies were administered in five cases, consisting of granulocyte transfusions during aplasia (*n* = 4) and arterial embolization (*n* = 1). After excluding the two deceased patients, the median duration of antifungal treatment following allo-HCST was 4 (3, 4, 5, 6, 7, 8, 9, 10, 11) mo.

Of the 14 patients, three underwent transplantation from a MUD, four from a MMUD, and six patients from a MSD. One patient had an MMRD. Hematopoietic cell transplantation–specific comorbidity index ranged from 1 to 3. Stem cell source was bone marrow in 10 cases and peripheral blood stem cells (PBSCs) in four patients. The median dose of CD34^+^ stem cells was 5.9 × 10^6^ cells per kg bodyweight. Conditioning regimen was sub-myeloablative in 13 patients and myeloablative reduced toxicity in one and combined in all cases busulfan and fludarabine (14/14). All patients received serotherapy (antithymocyte globulin [*n* = 5] or alemtuzumab [*n* = 9]). Graft-versus-host disease (GVHD) prophylaxis was based ciclosporin and mycophenolate mofetil in all but one patient who received ciclosporin only.

1-year overall survival (OS) was 86% (12/14). Two patients (patients 4 and 6) died after engraftment failure. Patient 4 who suffered from disseminated scedosporiosis received a MSD transplant 4 mo after the diagnosis of IMI. He presented a secondary graft failure 10 mo after transplant and was retransplanted from the same geno-identical donor 23 mo after the initial diagnosis of fungal infection and 9 mo after the first transplant. He died of a massive hemoptysis at day +10 after second HSCT. Patient 6 did not achieve engraftment and died of septic shock in relation with refractory *Lomentospora* fungemia at day +50 after HCST.

The median time of neutrophil engraftment and platelet recovery were 17 and 18 days after transplant, respectively. Among patients achieving engraftment, median dihydrorhodamine (DHR) at 1 mo after HCST reached 89%. At 3 mo, nine patients had donor chimerism above 95 % and three had mixed chimerism.

Posttransplant infections occurred in eight cases and were bacterial (*n* = 3), viral (*n* = 3), or fungal infections (*n* = 2). The two fungal infections were de novo, occurred within the first days of aplasia, and were, respectively, *Rhodotorula* fungemia and *L. prolificans* infection. In addition, patient eight had a grade two acute skin GVHD treated with corticosteroids, patient 11 a transitory pulmonary hypertension 1 year after allograft, and patient 12 experienced thrombotic microangiopathy that led to ciclosporin cessation.

All patients who presented aspergillosis with progression prior to allo-HSCT, including two localized and two disseminated infections, had a favorable clinical and radiological outcome. Moreover, all four patients who had IMI with PR demonstrated a constant improvement of radiological findings after HCST. At a median follow-up of 1 year, all surviving patients had a persistent clinical cure.

### Literature review (Table 3)

**Table 3.** Literature review of reported cases of CGD patients undergoing HSCT after an IMI diagnosis

Study (reference)	Age at the moment of HSCT	IMI	Localization	Outcome
(30)	​	​	​	Engraftment: Yes
*N* = 1	8 years	Aspergillosis with DP	Disseminated (lung, skin and muscular)	GVHD: No
				Cured of IMI and alive: Yes
(28)	​	​	​	Engraftment: Yes
*N* = 1	4 years	Aspergillosis with DP	Pneumonia and contiguous osteomyelitis	GVHD: grade III skin
				Cured and alive: Yes
(13)	​	​	​	Engraftment: 2/4
*N* = 4	MA: 15.5 years [14–16]	Four aspergillosis with DP	One disseminated infection including heart and bones involvement, two disseminated infections with lung and CNS involvement, one pneumonia	GVHD: 2/4
				Cured and alive: 1/4^a^
(13)	​	​	​	Engraftment: Yes
*N* = 1	20 years	*S. apiospermum* infection with DP	Disseminated (lung and paraspinal)	GVHD: No
				Cured and alive: no^b^
(31)	​	​	​	Engraftment: Yes
*N* = 1	18 years	*S. apiospermum* infection with DP	Paraspinal abscess	GVHD: No
				Cured and alive: No^c^
(32)	​	​	​	Engraftment: Yes
*N* = 1	8 years	Aspergillosis with DP	Disseminated (lung + vertebrae)	GVHD: grade III gut + liver
				Cured and alive: Yes
(33)	​	​	​	Engraftment: Yes^d^
*N* = 1	3 years	Aspergillosis with DP	Disseminated (lung + CNS)	GVHD: grade II
				Cured and alive: Yes
(34)	​	​	​	Engraftment: 7/9
*N* = 9	MA: 2 years [2–3]	Aspergillosis with CR	Lung	GVHD: 2/9
				Cured and alive: 6/9^e^
(35)	​	​	Two pneumonias	Engraftment: 2/3
*N* = 3	15, 17, and 21 years	Two aspergillosis with CR and one aspergillosis with DP	One disseminated infection (brain; kidney)	GVHD: 1/3 (grade 2 skin)
				Cured and alive: 2/3^f^
(36)	​	​	​	Engraftment: Yes
*N* = 1	6 years	Aspergillosis with CR	Pneumonia	GVHD: No
				Cured and alive: Yes
(37)	​	​	​	Engraftment: Yes
*N* = 1	13 years	Aspergillosis with CR	Sinusitis	GVHD: grade 2 liver
				Cured and alive: Yes
(38)	​	​	​	Engraftment: Yes
*N* = 1	8 years	Aspergillosis (no information about infection stage)	Pneumonia	GVHD: No
				Cured and alive: Yes
(39)	​	​	​	Engraftment: 2/2^g^
*N* = 2	13 and 5 years	Aspergillosis with DP	Pneumonia	GVHD: 1/2 (grade 2 skin)
				Cured and alive: 2/2
(40)	​	​	​	Engraftment: Yes^h^
*N* = 1	3 mo	Aspergillosis with DP	Pneumonia	GVHD: grade 2 skin
				Cured and alive: Yes
(23)	​	​	​	Engraftment: 11/11
*N* = 11	MA: 14 years [13–16]	*S. apiospermum* infection with DP	Two disseminated infections (one bones + lung; one CNS + lung), nine pneumonias	GVHD: 1/11
				Cured and alive: 10/11^i^
(29)	​	​	​	Engraftment: Yes
*N* = 1	12 years	Aspergillosis with DP	Pericarditis	GVHD: grade 2 GI
				Cured and alive: Yes
(29)	​	​	​	Engraftment: Yes
*N* = 1	26 years	Two aspergillosis with DP	Pneumonia with contiguous osteomyelitis	GVHD: grade 2 GI
				Cured and alive: Yes
(41)	​	​	​	Engraftment: 2/2^j^
*N* = 2	1 and 11 years	Aspergillosis with CR	One disseminated infection (no information about localization)1	GVHD: 0
				Cured and alive: 2/2
(42)	​	​	​	Engraftment: Yes
*N* = 1	5 years	Two scedosporiosis with SR^k^ and four aspergillosis with SR^k^	Pneumonia	GVHD: grade 2 GI
				Cured and alive: Yes
(43)	​	​	​	Engraftment: 13/13
*N* = 13	MA: 18 [5–25]	Seven aspergillosis with DP	Six pneumonias, five disseminated diseases, one spinal abscess, and one muscular abscess	GVHD: 4/13
				Cured and alive: 10/13
This paper	​	​	​	Engraftment: 12/14
*N* = 14	MA: 15 years [10–30]	See Table 1	See Table 1	GVHD: 1/14
				Cured and alive: 12/14

*N* = number of patients. CGD, chronic granulomatous disease; MA, median age; GI, gastrointestinal; CNS, central nervous system; BM, bone marrow.

a The causes of death were progression of aspergillosis before engraftment in two patients and a massive hemorrhage in one patient.

b The patient died from an uncontrolled hemorrhage through an eroded carotid artery.

c The patient succumbed to a complication of tracheostomy 73 days after transplantation.

d The patient had an HSCT failure following a first HSCT with reduced toxicity conditioning.

e One patient died from ARDS, another from systemic BK virus infection, the third from complications of chronic GVHD.

f One patient died 1 day prior to transplantation from multiorgan failure secondary to disseminated *A. nidulans* infection.

g One patient had HSCT failure following a first HSCT with myeloablative conditioning.

h The patient experienced an allograft rejection following a cord blood transplantation from an unrelated donor before her last HSCT.

i One patient with IPA died from a steroid-refractory acute GVHD of grade III–IV at day 125 after HSCT.

j One of the patient received a boost with a BM donor 37 days after HSCT.

k According to the definitions of the study published by Klin et al, the disease was stable if the patient had no clinical symptoms related to the disease, and pretransplant radiology revealed no changes to suggest progression at day 0.

The initial PubMed search retrieved 89 citations. After exclusion of duplicates and articles with titles and abstracts not meeting inclusion criteria, 36 full-text articles were assessed for eligibility. Two further articles were excluded and 34 were included, a majority of which were case reports.

Overall, the literature review included 71 patients. 61 (86%) were male. The majority (58/71, 82%) of patients had X-linked CGD, one (1%) was an X-linked carrier, and twelve (17%) patients had AR CGD. Median age at time of HCST was 11 years (1–17.5). Among the 69 IMI with available data on infection status, 18 (26%) were associated with CR, 9 (13%) with PR or SR, and 42 (61%) with DP at the time of HSCT. *Aspergillus* sp. was involved in 63 (89%) patients and represented approximately three quarters (32/39) of IMI with DP. Other molds recorded included *S. apiospermum* (6/71) and *L. prolificans* (1/71). Most patients had localized infections (40/69), mainly pneumonia (34/69) and paraspinal abscess (2/56). Conversely, 31 patients presented disseminated IMI, mainly caused by *Aspergillus spp* (24/31).

Donor HLA matching was distributed as follows: 25 MSD, 33 MUD, five MMRD, three MMUD, and three with missing data. Among 58 patients with information about stem cell source and conditioning, 43 patients were infused with BM, while the remaining patients received PBSC (14/58) and cord blood cells (1/58). Myeloablative conditioning (31/58), as defined by European Society for Blood and Marrow Transplantation (EBMT) classification, was the main conditioning used followed by sub-myeloablative reduced-toxicity conditioning (RIC) (16/58) and reduced intensity conditioning (11/58) (15). Engraftment was achieved in 61 (86%) patients, while 20 (28%) patients experienced GVHD. Clinical cure was obtained in 58 patients (82 %) and in 77% (40/52) of those with active IMI (DP or SR or PR). Among the 15 (21%) deaths, six (40%) were attributable to IMI progression and two (17%) were caused by GVHD. Eight out of 12 deaths were linked to IMI with DP.

## Discussion

In this study, we describe the clinical outcome of 14 CGD patients undergoing HCST after a diagnosis of IMI. The mortality rates of 14% (2/14) were comparable to previous reports including our own data from the French registry (22). The two reported deaths in our cohort were linked to graft failure (primary and secondary graft failure in one case each). Moreover, the rate of engraftment was 86 % in our study. Chiesa et al. found an 88% rate of engraftment among 712 patients undergoing HCST, while others authors reported engraftment rates ranging from 77% to 95% of patients (12, 23, 24, 25). More recently, Riller et al. found an engraftment rate of 80% among 65 patients transplanted from an HLA-haploidentical family donors (26). Of note, one of the patients with graft failure had a history of HLA-alloimmunization, which is at risk of engraftment failure (27).

Among the 10 patients with active IMI (DP or SR or PR) at the time of HCST, all patients with aspergillosis—including three with disseminated infections—were cured. These results are consistent with data from the literature showing that 26 CGD patients (mostly case reports) were successfully transplanted in the setting of active aspergillosis (sinusitis, pulmonary, paraspinal abscess, or disseminated infections) after initial failure of conventional antifungal treatment (23, 28, 29, 30, 32, 33, 37, 38, 39, 40, 41, 43). These results have led some authors to propose HCST as a therapeutic approach in refractory aspergillosis after 3 mo on antifungal treatment (41). The good survival and sustained clinical cure observed in all patients from our cohort with IMI in CR further reinforces previous evidence supporting the safety of performing HSCT in the context of resolved pulmonary aspergillosis (34, 35, 36, 42). Cerebral abscesses are particularly challenging. In our cohort and in the literature review, we identified two patients and six cases, respectively, with *Aspergillus* spp related CNS infections. Six of these eight patients (67%) achieved a favorable outcome after HSCT. The place of surgery before HSCT is a matter of debate. In the literature, surgery was performed in two cases of aspergillosis (to treat a pneumonia and a brain abscess in on case each) and in one patient with a *S. apiospermum* lung abscess. All three experienced favorable post-HSCT outcomes. Previous French CGD cohorts reported that surgery was part of infection management in 52% of 29 IMI episodes and in 55% of non-*Aspergillus* mold infections (44). The use of surgery was less frequent these recent years; we believe that surgery should not be systematically performed before HSCT and should always be balanced against the risk of delayed postoperative healing in CGD and the risk of postponing HSCT.

The indication for HSCT in patients with infections due to *S. apiospermum* and *L. prolificans* deserves careful discussion. These infections are frequently disseminated, as recently demonstrated in a recent analysis of cases of non-*Aspergillus* mold infections from the CEREDIH registry (44), which likely contribute to their poor prognosis. Published experience with HSCT in this context remains limited and showed conflicting results: Parta et al. reported a favorable outcome after haploidentical HSCT for therapy-refractory *S. apiospermum* pericarditis, and Kline et al. described two patients with pneumonia and disseminated *S. apiospermum* infection successfully transplanted 1 year after IMI diagnosis, whereas Seger et al. and Gompels et al. reported fatal outcomes after haploidentical HSCT in cases of disseminated or paraspinal therapy-refractory *S. apiospermum* infection (13, 31, 43). In both cases reported in this series, the patients had received pretransplant granulocyte transfusions, which may have contributed to graft rejection by triggering alloimmunization. The use of granulocyte transfusions in CGD remains controversial for this reason. Based on our experience, they should be reserved for infections truly refractory to optimal antifungal therapy and carefully weighed against the associated risk of alloimmunization.

Based on our experience and the literature, the decision to proceed with HSCT in the context of ongoing fungal infection should always be individualized and made jointly with infectious diseases specialists, hematologists, and immunologists. In patients showing complete, stable, or PR to antifungal therapy, we would not recommend delaying HSCT. In contrast, for patients with progressive disease, it appears reasonable to attempt HSCT as salvage treatment after 3 mo of optimized antifungal treatment, as previously suggested (41); however, at this stage, our data are too limited to support the use of this strategy for other mold infections.

### Conclusion

IMIs remain a serious infectious complication of CGD, highlighting the importance of primary antifungal prophylaxis in these patients. HCST should be discussed as a salvage therapy in CGD patients with aspergillosis and DP. The sustained clinical cure observed in patients undergoing HCST in the setting of aspergillosis with CR or PR provides reassuring data that should be validated in larger cohort.

## Materials and methods

### Patients and definitions

Using CEREDIH, we retrospectively identified all CGD patients who underwent HSCT between 2008 and 2022 in France. Among these 75 patients, we selected all CGD patients who underwent HSCT within 2 years after an IMI diagnosis. Episodes of IMI were defined according to the European Organization for Research and Treatment of Cancer (EORTC) and Mycoses Study Group Education and Research Consortium criteria (14). Only probable or proven IMI according to these criteria were included. We excluded patients who experienced IMI after HSCT.

The intensity of conditioning was defined in adult patients according to EBMT classifications (15), with the addition of RIC (busulfan = 9.6 mg/kg i.v.) and myeloablative reduced toxicity conditioning (busulfan = 12.8 mg/kg i.v.) (16, 17). In pediatric patients, RIC was defined by a reduced target of busulfan cumulative area under the curve (AUC) (60 mg × h/L). HLA compatibility with an adult related or unrelated donor was defined by high-to medium-resolution typing for HLA-A, B, -C, -DR, and -DQ loci. Primary HCST failure was defined as failure to achieve neutrophil recovery or donor chimerism <5% on day +28 after HSCT. Chimerism was analyzed on whole blood by quantitative real-time polymerase chain reaction for insertion/deletion polymorphism as previously described (18). Phagocyte oxidative burst was assessed by DHR test. According to EBMT/Center for International Blood and Marrow Transplant Research definitions, time to resolution of aplasia was defined by the first of three successive days with an absolute neutrophil count of ≥500/μl. Time of platelet recovery was defined as the first of three consecutive days with a platelet count of 20,000/μl or higher in the absence of platelet transfusion for seven consecutive days (19). Grading of acute GVHD was performed according to the Glucksberg-Seattle criteria (20).

IMIs were classified according to Segal et al. criteria at the time of HCST: CR was defined as survival and resolution of all attributable symptoms and signs of disease as well as resolution of radiological lesions and documented clearance of infected sites that are accessible to repeated sampling; PR was defined as survival and improvement of attributable symptoms and signs of disease, improvement in radiological lesions, and documented clearance of infected sites that are accessible to repeated sampling; SR was defined as survival and stability of all attributable symptoms and signs of disease, radiological stabilization, or persistently positive results of specimens from infected sites, and DP was defined as worsening clinical symptoms or signs of disease, new sites of disease or radiological worsening of preexisting lesions, or persistently positive results of specimens from infected sites. (21). The delay between IMI and HCST was quantified according to the date of microbiological diagnosis of IMI and the last allograft received.

### Study objectives

The primary objective was to describe 1-year OS. The secondary objectives were to assess the rate of HSCT failure, DHR normalization, GVHD, and rate of persistent clinical and radiological cure of IMI at 1 year.

### Data collection

Data were recorded on a standardized form and included sex, age at CGD diagnosis, genetic pattern of inheritance (AR or X-linked CGD), comorbidities such as chronic kidney disease or alloimmunization, age at time of IMI, history of inflammatory disease, use of immunosuppressive treatments in the year prior to IMI diagnosis, infection status at the time of HCST, date of IMI, fungal species, site of infection, date of HCST, donor type, CD34 cells count, treatment degree of HLA match, stem cell source, conditioning intensity, conditioning regimen, dose of busulfan, GVHD prophylaxis, engraftment, time of resolution of aplasia, time of platelet transfusion free, 3-mo chimerism status, post-HCST infection, complication related to HSCT, DHR test at 1 mo after HSCT, 3-mo clinical and radiological evaluation, 1-year OS, and clinical and radiological cure evaluation at 1 year after HCST.

### Literature review

In addition, we reviewed the literature from 1998 to 2025 with PubMed for published cases of HSCT performed in the setting of IMI. The following keywords were used: hematopoietic stem cell transplantation, IMIs, chronic granulomatous disease, aspergillosis, mucormycosis, scedosporiosis, lomentosporiosis, refractory fungal infections, and post allograft infection. Articles were selected for review if their titles or abstracts suggested that they reported individual or group data from CGD patients who underwent HCST in the setting of IMI. References from relevant articles were screened for additional cases. We excluded duplicate cases and cases without individual case details. Articles were excluded if patients did not meet the EORTC criteria for proven or probable IMI or if IMI occurred after HCST.

### Statistical analyses

Continuous variables are presented as median and interquartile range, and categorical variables as number and percentage.

### Online supplemental material

Main characteristics of CGD patients undergoing HCST with or without prior IMI are summarized in Table S1.

## Ethics

In accordance with French regulatory requirements, we obtained informed consent from patients and/or parents upon registration in the CEREDIH registry.

## Supplementary Material

Table S1 shows the characteristics of 75 CGD patients who had HSCT according to “prior IMI in last 2 years” status.

## Data Availability

The data underlying this study are not publicly available due to patient privacy reasons. The data are available upon reasonable request to the corresponding author: fanny.lanternier@aphp.fr.
