# Supplementary material for: Hematopoietic stem cell transplantation following invasive mold infection in chronic granulomatous disease: Insights from a case series and literature review
Source: J Hum Immun. 2026 Mar 5;2(3):e20250164. doi: 10.70962/jhi.20250164 (PMC13177795; doi:10.70962/jhi.20250164)
Supplement: Table S1 — shows the characteristics of 75 CGD patients who had HSCT according to “prior IMI in last 2 years” status. [file jhi_20250164_tables1.docx]

Supplementary data

Characteristics of 75 CGD patients who had HSCT according to “prior IMI in last 2 years” status

| Age at the moment of HCST [IQR] (years)  Donor type  MSD  MUD/MMUD  MMRD  1 year-Mortality | HCST with IMI  N=14 (%)  15 [10-30]  6 (43)  7 (50)  1 (7)  2 (14) |  | HCST without IMI  N=61 (%)  9 [4-15]  18 (30)  35 (57)  8 (13)  8 (13) |
| --- | --- | --- | --- |

Abbreviation : CGD : chronic granulomatous disease, HSCT : hematopoietic stem cell transplantation [IQR] : interquartile range, MSD : matched sibling donors, MUD : matched unrelated donors , MMRD : mismatched related haploidentical donors
